# Laparoscopy Versus Open Technique for Inguinal Hernia Repair in Children: A Systematic Review and Meta-Analysis

**DOI:** 10.7759/cureus.74992

**Published:** 2024-12-02

**Authors:** Diogo S Almeida, Luís Henrique A Medina, Nathalia C Miranda, Tiago S Almeida, Letícia F Mattos, Maria E Marchi, Marina M Azaro, Gabriel Q Gousseaud, Elaine R Coelho

**Affiliations:** 1 General Surgery, Bahiana School of Medicine and Public Health, Salvador, BRA; 2 General Surgery, Zarns Health Education Institute, Salvador, BRA; 3 Digestive System Surgery, Roberto Santos General Hospital, Salvador, BRA

**Keywords:** inguinal hernia repair, inguinal hernia surgery, laparoscopic hernia repair, laparoscopic pediatric surgery, open inguinal hernia repair

## Abstract

This study aims to compare operative time, recurrence, and complications between laparoscopic and open techniques for the repair of inguinal hernia in children. Pubmed and Embase databases were systematically searched for studies of pediatric patients who underwent open or laparoscopic inguinal hernia procedures. The main outcomes were operative time, recurrence, and complications. Statistical analysis was performed using RevMan 5.4.1 software (The Cochrane Collaboration, Copenhagen), and heterogeneity was assessed using the I² strategy. Four randomized clinical trials were included in the statistical analysis, totaling 662 patients, with 337 patients in the laparoscopic technique group. Operative time was significantly shorter in the laparoscopic technique group (STD -0.75, 95% CI (-1.43, -0.07), P < 0.00001, I² = 92%). Recurrence (RR 0.60, 95% CI (0.18, 2.02), P = 0.54, I² = 0%) and complications (RR 0.49, 95% CI (0.12, 2.03), P = 0.32, I² = 67%) were statistically similar between the two groups. The laparoscopic technique for inguinal hernia repair in children proved to be faster, with similar recurrence and complication rates compared to the open technique.

## Introduction and background

Indirect inguinal herniorrhaphy is one of the most frequently performed surgical procedures in children. The overall incidence of inguinal hernias in childhood ranges from 0.8% to 4.4%, is up to 10 times higher in boys than in girls, and premature infants are more affected [1]. The vast majority of inguinal hernias in infants and children are indirect, resulting from a persistent patent processus vaginalis [2]. The treatment of this pathology is especially important in the pediatric population due to its high prevalence and risk of severe complications, such as incarceration and strangulation, particularly in infants and premature babies [1].

Open repair is the most traditional and gold standard approach in the surgical management of pediatric inguinal hernia, demonstrating low complication rates, between 1.1% and 1.2% [3]. This approach can be performed using various techniques such as external ring incision, hernial sac twisting, and single or double ligation [3,4]. The literature highlights the high ligation technique of the hernial sac, used in the largest series reported by a single surgeon [5]. Nevertheless, there is no international consensus regarding the ideal technique [3].

Conversely, laparoscopic intervention began in the 1990s and offers advantages such as easy visualization of the contralateral internal inguinal ring, avoids the small risk of incarceration of a metachronous hernia as well as the cost and anxiety of a second operation, lower risk of ascending testis, and reduced risk of injury to the vas deferens and vessels [1,3,6]. The percentage of pediatric surgeons using laparoscopic exploration is increasing. There are two basic techniques: purely intracorporeal ligation and laparoscopic-assisted extracorporeal ligation, with the latter being associated with a higher recurrence rate compared to the intracorporeal and open techniques [7]. The downside of this approach is that laparoscopic exploration cannot differentiate between a patent processus vaginalis and a true hernia. A study showed that laparoscopic inguinal hernia repair was associated with a lower incidence of metachronous contralateral hernias compared to open inguinal hernia repair and a higher incidence of recurrence [8].

Another debate about the subject is the necessity of contralateral exploration; it involves a choice between treating only obvious hernias versus preventing metachronous hernias by closing any patent processus vaginalis that is found. In the 1950s, reports by Duckett and Rothenberg led to routine contralateral exploration based on a 30% incidence of contralateral hernias. However, this open exploration was reconsidered in the 1990s due to associated risks, such as infertility. Presently, most surgeons avoid routine contralateral exploration in open repairs [1]. Laparoscopy offers a middle ground, enabling contralateral evaluation with minimal risk to the vas deferens and vessels [8].

Although the classic open inguinal hernia repair remains the gold standard for most pediatric surgeons, laparoscopic repair is being performed in many centers and is associated with lower complication rates [1,9]. The comparison between open and laparoscopic techniques shows similar recurrence rates, surgical time, and complications [3]. Therefore, a more comprehensive analysis of scientific evidence is necessary for laparoscopy to be considered superior to the gold standard, that is, the open technique.

The latest meta-analysis on the topic demonstrated that laparoscopic repairs generally result in fewer issues with wound appearance, fewer recurrences, fewer postoperative complications, and better wound scores compared to open repairs. However, the authors highlighted the use of small sample sizes in many of the studies [10].

Therefore, this meta-analysis aimed to compare the outcomes of laparoscopic and open-surgical approaches for pediatric inguinal hernia repair. By synthesizing data from randomized clinical trials, the research sought to evaluate key factors such as recurrence rates, complication rates, and postoperative recovery. The findings contribute to the evolving field of pediatric surgery, offering data with a higher degree of scientific evidence.

## Review

Materials and methods

This systematic review was conducted by selecting only randomized clinical trials that compared laparoscopic and open techniques for inguinal hernia repair in children. The articles were analyzed, and those that did not fit the PICOTT question and inclusion criteria were excluded. The present systematic review and meta-analysis adhere to the PRISMA guidelines.

The PICOTT question was formulated in the following manner: This review targets the pediatric population diagnosed with inguinal hernia, whose age varies from newborn to adolescent. It examines laparoscopic inguinal hernia repair as the primary intervention in comparison to the conventional open repair technique. The primary outcomes assessed include operative time, recurrence, and complications. The selected study design is a systematic review and meta-analysis, incorporating studies with adequate follow-up periods to ensure a comprehensive evaluation of these surgical outcomes.

Search Strategy

Pubmed and EMBASE were searched systematically from inception to June 2024 using the search strategy: (infants OR Children OR Pediatric OR "Pre term" ) AND ("inguinal hernia repair" OR "Inguinal mesh repair"). The protocol for this systematic review and meta-analysis was registered on PROSPERO (registration number: CRD42024598044).

Study Selection and Data Extraction

Two authors (MM and TA) conducted an independent and blinded search. Studies were selected for full-text reading based on the inclusion criteria. Conference abstract-only and observational studies were removed. Articles not available in the English language were also excluded. Disagreements were solved by consensus. 

Two researchers (DA and LM) extracted the data, which was then reviewed by a third author NM. The outcomes of interest were operative time, complication rate, and hernia recurrence. 

Assessment of Quality of the Studies 

The risk of bias was assessed using the Cochrane Collaboration tool. A study was classified as having a high risk of bias if one or more domains in the Cochrane Collaboration tool indicated a high or unclear risk. 

Statistical Analysis

Categorical outcomes (recurrence and complications) were expressed using a risk ratio with 95% CI. Continuous variables (operative time) were analyzed using the standardized mean difference. Data originally reported as median and interquartile range were converted to mean and standard deviation.

The studies that did not explicitly report key information used to analyze those outcomes in their tables were handled by active search in results presented by the text. 

The DerSimonian and Laird random-effects model was applied. Heterogeneity was assessed using the I² statistic, considering I² <25% (low heterogeneity), I² 25-50% (moderate heterogeneity), I² 50-75% (substantial heterogeneity), and I² >75% (high heterogeneity).

Statistical analysis was performed using Review Manager software, version 5.4.1 (The Cochrane Collaboration, Copenhagen).

Results

Study Selection

**Figure 1:**
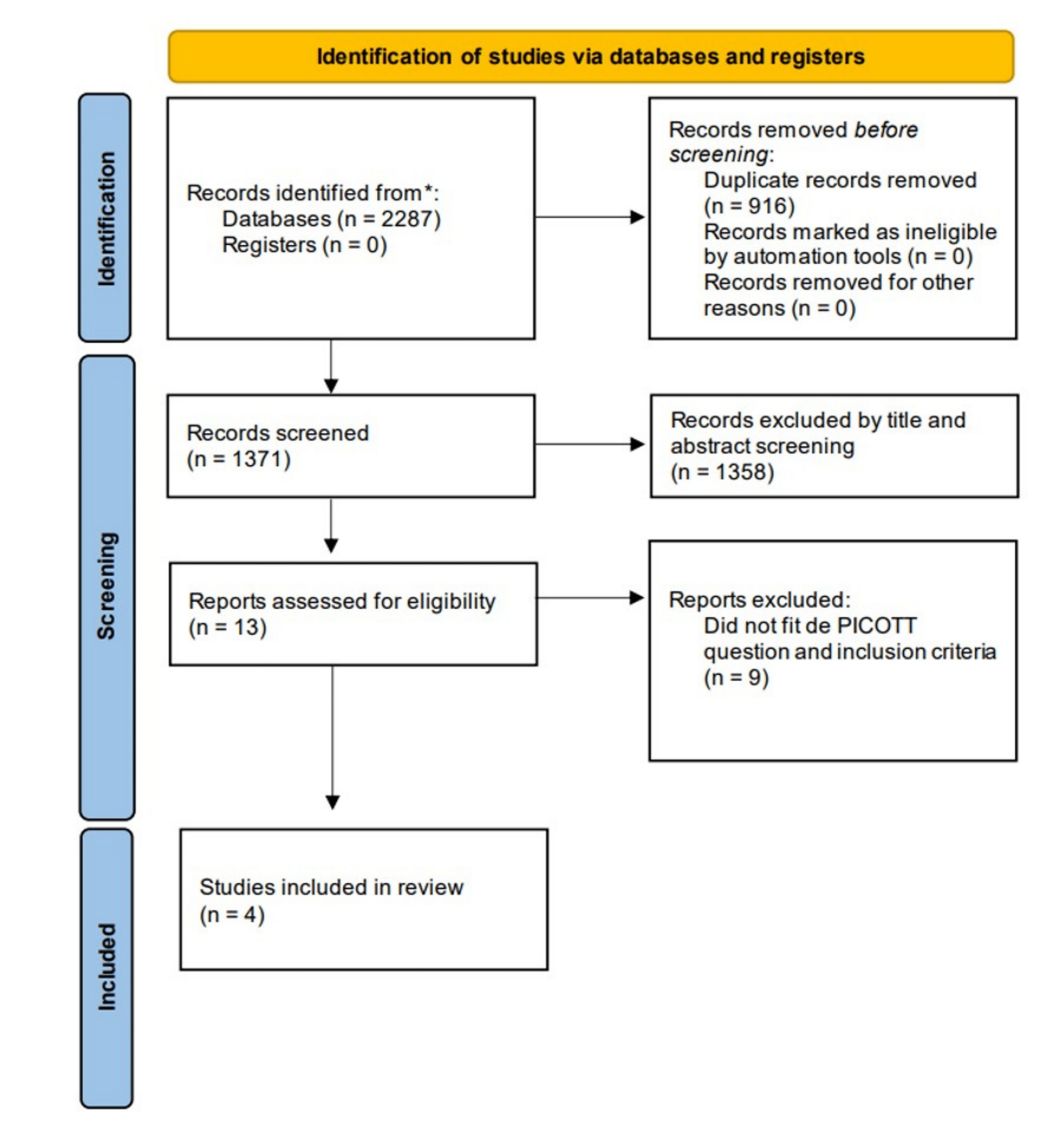
PRISMA 2020 flow diagram.

Through a preliminary database search, 2287 studies were obtained. After removing duplicates with the help of the Rayyan program, 1882 titles and abstracts were screened, leaving 13 studies for full-text evaluation. As shown in the flowchart (Figure 1), only four manuscripts fulfilled the eligibility criteria of this meta-analysis, all randomized controlled trials (RCTs).

Study Characteristics

**Figure 2:**
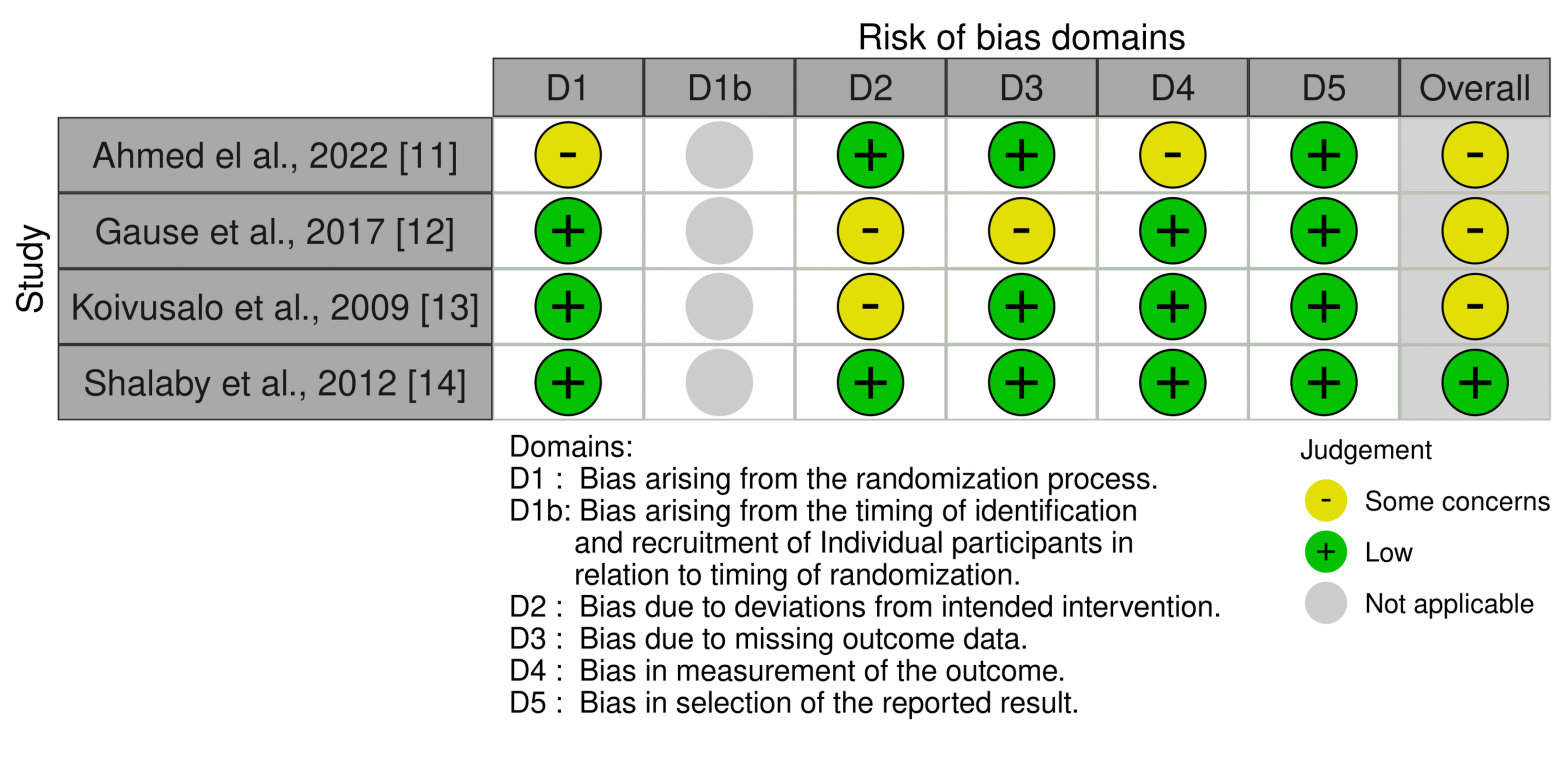
Risk of bias (RoB 2) summary for randomized studies.

Four RCTs were included in this analysis, as presented in Table 1. The studies varied in sample size, with a range of 41 to 296 patients. In all trials, a subset of patients underwent laparoscopic procedures, while others received open surgery. The average age differed by study and surgical approach, ranging from 0.08 years (Shalaby, 2012) to 6.1 years (Koivusalo, 2009) for laparoscopic and open methods. The overall risk of bias was rated as “some concerns” in three studies (Gause, 2017; Koivusalo, 2009; Ahmed, 2022) and “low” in one (Shalaby, 2012) as presented in Figure 2.

**Table 1:** Main characteristics of included studies. LH: laparoscopic herniorrhaphy; OH: open herniorrhaphy

Study	Design	Sample size	Laparoscopic	Age (years) LH	Age (years) OH	Gender (LH)	Gender (OH)	Overall risk of bias		Main findings	
						Male %	Male %				
Ahmed et al., 2022 [11]	RCT	296	148	1.0-13	1.0-15	89.86	89.8	Some concerns		LH technique is better than OH in terms of lower incidence of contralateral metachronous hernia, operative time, and recurrence rate.	
Gause et al., 2017 [12]	RCT	41	26	1.03	0.54	73	80	Some concerns		OH and LH both appear to be equally safe and effective, LH demonstrated shorter operative times	
Koivusalo et al., 2009 [13]	RCT	89	47	0.65-15	1.6-15	76	71.4	Some concerns		LH offers no advantages over OH of pediatric IH.	
Shalaby et al., 2012 [14]	RCT	250	125	0.08-2	0.08-2	30.4	73.6	Low		LH showed less iatrogenic testicular ascension, better cosmetic results, and reduced operative time	

Outcomes 

It was observed that operative time was significantly shorter in the laparoscopic group (standard mean difference (SMD) -0.75, 95% CI (-1.43, -0.07), P = 0.03, I² = 93%). Additionally, the recurrence rate (RR 0.50, 95% CI (0.14, 1.85), P = 0.44, I² = 0%) and complication rate (RR 0.38, 95% CI (0.11, 1.31), P = 0.09, I² = 53%) were statistically similar between the two groups.

Meta-Analysis

**Figure 3:**
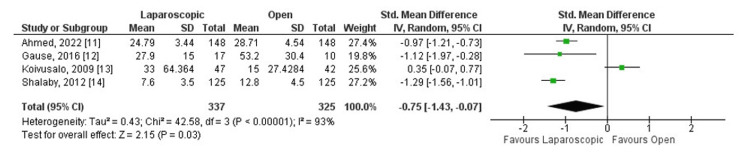
Laparoscopic repair versus open repair comparing operative time.

**Figure 4:**
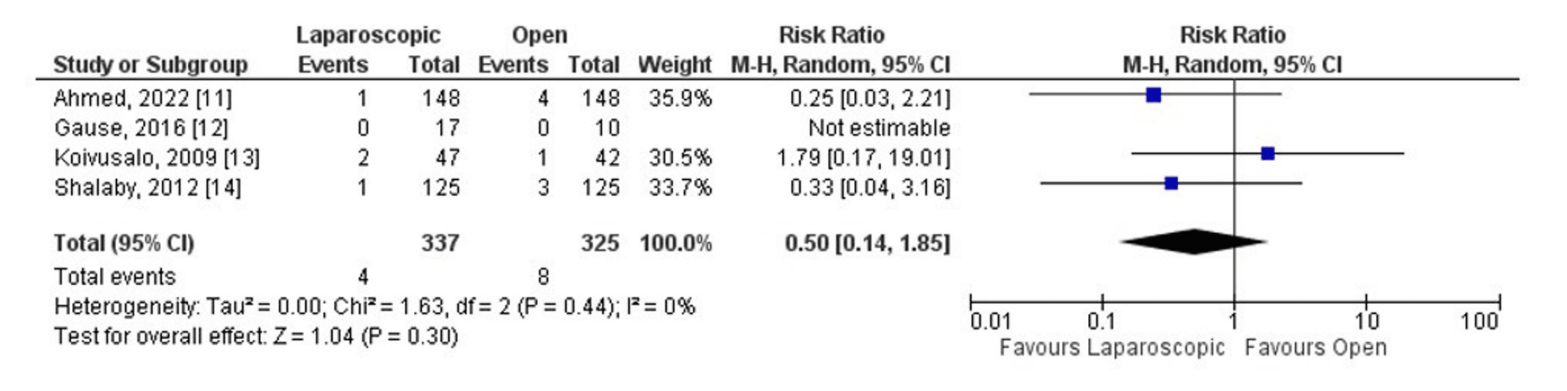
Laparoscopic repair versus open repair comparing recurrence.

**Figure 5:**
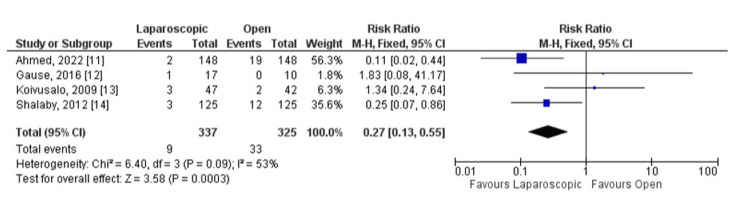
Laparoscopic repair versus open repair comparing complications.

All studies reported operative time, recurrence, and complications as outcomes (Figures 3, 4, 5). Considering the operative time, the SMD calculated was -0.75, 95% CI (-1.43, -0.07). The overall SMD is negative, suggesting that laparoscopic surgery tends to have a shorter operative time compared to open surgery. The 95% CI does not cross 0 (-1.43 to -0.07), indicating a statistically significant difference (p = 0.03). 

However, there was no statistically significant difference between groups for recurrence. The risk ratio calculated was 0.50, 95% CI (0.14, 1.85). The overall RR is less than 1, suggesting a lower risk of recurrence with laparoscopic surgery. However, the wide CI crossing 1 (0.14 to 1.85) indicates no statistically significant difference (p = 0.44).

Likewise, there was no statistically significant difference between the groups in terms of complications, including recurrence. The risk ratio calculated was 0.38, 95% CI (0.11, 1.31). The overall RR is less than 1, suggesting that laparoscopic surgery may have a lower risk of complications compared to open surgery. However, the wide CI crossing 1 (0.11 to 1.31) indicates that this result is not statistically significant (p = 0.09).

In the meta-analysis, heterogeneity varied between the outcomes analyzed. Looking at operative time, it was I² = 93%, indicating a substantial heterogeneity among the studies. For recurrence, it was I² = 0%, demonstrating no observed heterogeneity among the studies. Finally, the heterogeneity calculated for complications was I² = 53%, indicating a moderate heterogeneity between the studies analyzed.

Discussion

Clinical Outcomes 

Our analysis demonstrates that patients who underwent laparoscopic repair had a significantly shorter operative time compared with those who underwent open repair, while recurrence and complication rates were similar between the two groups. 

All studies brought recurrence rate as an outcome [11-14]. However, just three studies described recurrence events in their groups [11,13,14]. Koivusalo et al. had two recurrence events described in the laparoscopic arm and one described in the open arm, representing the only paper analyzed that numerically had more recurrence events in the laparoscopic group. Both Shalaby et al. and Ahmed et al. had one recurrence event described in the laparoscopic arm, differentiating in the number observed in the open group: Shalaby et al. with three events and Ahmed et al. with four events. Numerically, more recurrence events were found in the open group but not statistically significant.

Analyzing operative time, only Koivusalo et al. described laparoscopic repair as slower than open technique for the inguinal hernia repair. This study represented 25.1% of the population analyzed, and the duration of anesthesia in the laparoscopic group was considerably longer [13].

Complications were also described by all papers [11-15]. Koivusalo et al. and Gause et al. both had more complications in the laparoscopic group. However, different criteria were defined in the two studies. Koivusalo et al. described three events in the laparoscopic group, with postoperative pain as the criterion adopted, while Gause et al. described one event, analyzing wound infection and postoperative bleeding as the criteria. Dreuning et al. noted only operative complications (such as injury to the spermatic vessels or cord, tubal lesions, and bleeding) in the laparoscopic group, while general complications showed no differences between the groups, as was observed in our meta-analysis.

The other two studies that analyzed complications had more events described in the open arm, representing 62.8% of the population analyzed [11,14]. Shalaby et al. described 12 events in the open group, with hydrocele and unsightly scar as the criteria adopted. Ahmed et al. described 19 events, considering metachronous contralateral hernia as the criteria for complications. Then, the complication rate was higher, but not statistically significant, in the open arm, considering the multiple criteria adopted by each paper. 

Comparison With Previous Reviews

A previous meta-analysis evaluated cosmesis and other postoperative complications in laparoscopic versus open pediatric inguinal hernia repair. However, it also included observational, prospective, and retrospective studies, which increases data heterogeneity and introduces a higher risk of bias in the data used [10].

Analyzing the operative time between the two techniques, the most recent systematic review on the subject demonstrated that this factor is primarily influenced by the presentation of the hernia. When the hernia is bilateral, the laparoscopic herniorrhaphy approach proves to be more advantageous, as it allows for the ligation of the contralateral hernia sac immediately after the laparoscopic correction of the first side, in contrast, to open herniorrhaphy, which requires the complete repetition of procedures on both sides [16]. This same standard was observed in the results of the present meta-analysis; the clinical trials involving bilateral hernias reported a shorter operative time for the laparoscopic approach compared to the open approach [11,12,14]. In addition, the only study that included unilateral hernias alone reported a longer operative time for the laparoscopic technique [13]. Finally, the lack of standardization across studies regarding the definition of operative time may influence the observed results.

According to our results, the open correction technique presented a higher number of recurrences compared to other techniques, although this difference was not statistically significant. Similarly, the study by Esposito et al. and Dreuning et al. also did not identify many differences in recurrence rates between the laparoscopic and open techniques [15,17]. Regarding complications, Esposito et al. reported a higher incidence of complications associated with the open technique, particularly infections. Our results support this observation, showing complications in both techniques, but with a higher prevalence in the open technique. This can be explained by the fact that the studies by Shalaby et al. and Ahmed et al. included a significantly larger number of patients in their samples. Furthermore, according to the results of the Liu et al. study, the rate of postoperative complications was higher in the group that underwent the open technique, when compared to patients who underwent the laparoscopic technique. This explains how laparoscopic herniorrhaphy has a fundamental role in reducing major postoperative complications [18].

Limitations of the Study

The limitations of this study include the limited number of available articles. Additionally, we were unable to differentiate between unilateral and bilateral inguinal hernia surgeries in our results, which may influence operative time outcomes. Furthermore, we observed significant heterogeneity (>25%) among studies when analyzing operative time and complication rates. Finally, we were unable to perform subgroup and sensitivity analyses due to the small number of included studies.

## Conclusions

In conclusion, it was observed that the repair of inguinal hernias via laparoscopic and open techniques in the pediatric population had similar complication and recurrence rates. However, the laparoscopic approach proved to be significantly faster, with shorter surgical time. More recent randomized clinical trials comparing the two surgical techniques may help highlight the advantages of using laparoscopy, considering the evolution and improvement of this technique in recent years.
